# A Novel Synthetic UV-Curable Fluorinated Siloxane Resin for Low Surface Energy Coating

**DOI:** 10.3390/polym10090979

**Published:** 2018-09-03

**Authors:** Chunfang Zhu, Haitao Yang, Hongbo Liang, Zhengyue Wang, Jun Dong, Lei Xiong, Jianping Zhou, Junjun Ke, Xi Xu, Weixian Xi

**Affiliations:** 1 School of Material Science and Engineering, Nanchang Hangkong University, Nanchang 330063, China; Zhuchunfang_123@163.com (C.Z.); yht@nchu.edu.cn (H.Y.); 18270890654@163.com (Z.W.); dj303@126.com (J.D.); xionglei@nchu.edu.cn (L.X.); zf161162@163.com (J.Z.); 18779160082@163.com (J.K.); xuxi19930904@163.com (X.X.); 2 Department of Chemical and Biomolecular Engineering, University of California Los Angeles, Los Angeles, CA 90095, USA; 3 Department of Orthopaedic Surgery, University of California Los Angeles, Los Angeles, CA 90095, USA

**Keywords:** ultraviolet (UV) curable coatings, low surface energy materials, fluorinated siloxane resin

## Abstract

Low surface energy materials have attracted much attention due to their properties and various applications. In this work, we synthesized and characterized a series of ultraviolet (UV)-curable fluorinated siloxane polymers with various fluorinated acrylates—hexafluorobutyl acrylate, dodecafluoroheptyl acrylate, and trifluorooctyl methacrylate—grafted onto a hydrogen-containing poly(dimethylsiloxane) backbone. The structures of the fluorinated siloxane polymers were measured and confirmed by proton nuclear magnetic resonance and Fourier transform infrared spectroscopy. Then the polymers were used as surface modifiers of UV-curable commercial polyurethane (DR-U356) at different concentrations (1, 2, 3, 4, 5, and 10 wt %). Among three formulations of these fluorinated siloxane polymers modified with DR-U356, hydrophobic states (91°, 92°, and 98°) were obtained at low concentrations (1 wt %). The DR-U356 resin is only in the hydrophilic state at 59.41°. The fluorine and siloxane element contents were investigated by X-ray photoelectron spectroscopy and the results indicated that the fluorinated and siloxane elements were liable to migrate to the surface of resins. The results of the friction recovering assays showed that the recorded contact angles of the series of fluorinated siloxane resins were higher than the original values after the friction-annealing progressing.

## 1. Introduction

Low surface energy materials (LSEMs) have been widely investigated due to their self-cleaning, drag-reducing, stain-resisting, and anti-fouling properties, which have enabled LSEMs to be applied as food packaging, and protective or anti-adhesion coatings [1,2]. In the low surface energy coating fabrication process, the two most significant factors are chemical structure and appropriate surface roughness [3,4]. For the former, researchers usually use –CF_3_ and –CF_2_ functional groups to create low surface energy of ~15 mN·m^−1^ and ~25 mN·m^−1^, respectively [3]. However, the extensive use of fluorocarbon resins is limited by poor compatibility, environmental contamination, bioaccumulation, and toxicity issues [3,4,5,6,7]. Therefore, to overcome this limitation, in this work, we proposed that the introduction of siloxane could increase the compatibility of the polymer.

In numerous organo-silicon compounds, polysiloxane and siloxane have been frequently investigated and reported due to their low-surface energy [8]. Polysiloxane not only exhibits thermal stability, low-surface energy, and chemical resistance, but also has a lower glass transition temperature, as well as non-toxic and anti-biological attachment characteristics [9]. Zhan et al. [10] proposed using polysiloxane mixed with polycaprolactone as the soft segment, isophorone diisocyanate (IPDI) as the hard segment, and a siloxane coupling agent as the chain extender to synthesize the silicone-modified polyurethane. They demonstrated that the increase in organo-silicone compound concentration led to the increase in the contact angle of the material surface. Li et al. [9] used α,ω-hydroxy-terminated polydimethylsiloxane (HTPDMS’ hydroxyl value ≥8%) and methyltrimethoxysilane with a dibutyl tin dilaurate (DBTL) catalyst to create a superhydrophobic surface.

The copolymer consists of fluorinated polydimethylsiloxane segments that might yield high performance materials. Yan et al. [11], using hydroxy-terminated polydimethylsiloxane, dicarboxyl terminated poly(2,2,3,4,4,4-hexafluorobutyl acrylate) (CTHFA), toluene diisocyanate, and 2-hydroxyethyl methacrylate (HEMA), produced a new ultraviolet (UV)-cured vinyl-terminal fluorinated siloxane graft copolymer (Vi-PFSi). The coating surface energy decreased from 45.52 mN/m^2^ to 15.40 mN/m^2^ when the concentration of the Vi-PFSi was loaded at 0.5 wt %. In this case, the hydrogen-containing polydimethylsiloxane was used as a functional spacer.

Based on the characteristics of the organo-silicon compound, here we designed and synthesized three types of fluorinated acrylate on organo-silicon [12,13,14]. The copolymer was synthesized from hydropolydimethylsiloxane, fluorinated acrylate, mercaptoethanol, acrylic acid, and 3-isocyanatomethyl-3,5,5-trimethylcyclohexyl isocyanate (IPDI) [15,16], which was then grafted to UV-cured fluoro-silicone resin. The surface properties of the modified UV-curable polyurethane were examined by contact angle measurements with water. The surface element contents were evaluated by X-ray photoelectron spectroscopy (XPS). In addition, the acid and alkali resistance were also evaluated in 10 wt % NaOH and 10 wt % H_2_SO_4_ solution. Our results indicate that this modified resin’s chemical resistance and physical properties were significantly improved with 1 wt % modification with synthesized fluorosilicone. The novelty of this work was that the friction recovering assays showed that the series of fluorinated siloxane resin’s contact angles recovered to higher than the original values after the friction-annealing progressing, making it a huge application prospect.

## 2. Materials and Experiment

### 2.1. Materials

Acrylic acid (AA; purity ≥ 99.8%) was purchased from Xilong Chemical Co., Ltd. (Shantou, China). Allyl glycidyl ether (AGE; purity ≥ 99.5%) and mercaptoethanol (≥99.5%) were purchased from Aladdin Co. Isophorone diisocyanate (IPDI; Bayer Reagents Co., Ltd., Shenzhen, China), hexafluorobutyl acrylate (HFOA; represented by G01 in this article), dodecafluoroheptyl acrylate (DFOA; referred to as G05 in this article), and trifluorooctyl methacrylate (TFOMA; referred to as G06B in this article; purity ≥ 99.5%) were obtained from Harbin Sunshine Fluorine Silicon Chemical Co., Ltd., Harbin, China). Hydrogen-containing polydimethylsiloxane (H-PDMS; purity ≥ 99%) was purchased from Shenzhen Chonghuaxin Technology Co., Ltd. (Harbin, China). DR-U356 was obtained from Changxin Material Industry Limited by Share Ltd. (Harbin, China).

### 2.2. Synthesis of Monomers

#### 2.2.1. Synthesis of Hydroxyl-Containing Polydimethylsiloxane (OH-PDMS)

**Figure 1:**
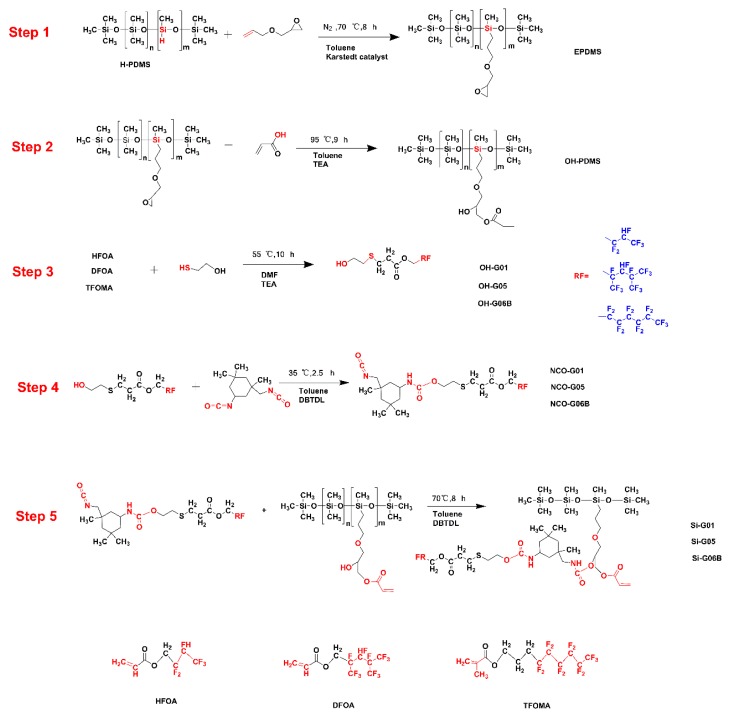
Synthesis of novel ultraviolet (UV)-curable fluorinated siloxane resins.

The hydroxyl-containing polydimethylsiloxane was prepared via a two-step reaction. Firstly, 0.15 mol (17.1 g) allyl glycidyl ether (AGE), an equal mass of toluene, and 300 ppm of Kastedt’s catalyst (according to the total mass of reactants) were added to a three-neck, round-bottom flask, heated to 65 (protected by N_2_). Then, the 50 g H-PDMS were slowly dropped into the bottom while the temperature was increased to 70 °C (6–8 h). Vacuum distillation was used to remove extra AEG and toluene to obtain the epoxy polydimethylsiloxane (EPDMS) (Step 1, Figure 1). Secondly, 15 g EPDMS and the same mass of toluene were added to the three-neck, round-bottom flask, the mixture was heated to 75 °C and held for 30 min. After that, 0.0021 mol AA, 1 wt % (the total mass of the reaction system) P-hydroxyanisole (MEHQ) and 0.413 g *N*,*N*-dimethyl benzyl ammonia (DBMA) were dropped into the bottom, and the mixture was heated to 95 °C. NaOH- and NaCl-saturated solutions were used to wash extra AA and MEHQ to obtain the hydroxyl-containing polydimethylsiloxane (OH-PDMS) (Step 2, Figure 1).

#### 2.2.2. Modification of Fluorinated Intermediaries (OH-G01, OH-G05, and OH-G06B)

Using the synthesis of OH-G01 as an example, 0.01 mol fluorinated monomer hexafluorobutyl acrylate (HFOA) and the same mass of dimethylformamide (DMF) were added into a three-neck, round-bottom flask, the temperature was increased to 55 °C, and 0.02 mol mercaptoethanol mixed with 0.5 wt % triethylamine solution were dripped into the bottom over 30 min. DMF and trimethylamine were removed after the reaction finished. OH-G05 and OH-G06 were synthesized using the same method (Step 3, Figure 1).

#### 2.2.3. Synthesis of Graft Fluorinated Siloxane Polymers (Si-G01, Si-G05, and Si-G06B)

Next, 0.01 mol IPDI, 0.01 mol fluorine intermediaries, and the same mass of toluene with the catalyst of 5 wt % DBTDL were added to the three-neck, round-bottom flask with magnetic stirring kept for about 2.5 h (Step 4, Figure 1). When the reaction was accomplished, a mixture of 0.01 mol hydroxyl-containing polydimethylsiloxane (OH-PDMS), an equal mass of toluene, and 5 wt % DBTDL were dropped into the flask slowly to obtain the Si-G01, Si-G05, and Si-G06B polymers (Step 5, Figure 1).

### 2.3. Preparation of Samples

#### 2.3.1. Resin Formulation

The UV-cured coatings were prepared as follows: the synthesized Si-G01, Si-G05, and Si-G06B, and the same acetone and initiator (Irgacure 1173) were added to the matrix resin (DR-U356) to study the influence of the properties of the contents of the different additives on the matrix resin (Table 1).

#### 2.3.2. Preparation Method

A tin plate (100 × 50 × 0.2 mm) was polished with sandpaper and cleaned using alcohol. Using the wire rod applicator (Tianjin Zhongya Material Testing Machine Factory, Tianjin, China) to prepare the coating, the width of the wire rod was 80 µm. The specific operation was as follows: a glass rod was used to dip the resin onto the tin plate, and the wire rod was pulled from one end of the tin plate to the other end.

### 2.4. Characterization

Proton nuclear magnetic resonance (^1^H-NMR) was tested using an UAVANCEIII 400 MHZ, manufactured by Agilent (Santa Clara, CA, USA); CDCl_3_ was used as the solvent. ^1^H-NMR was also used to measure the H content of the hydrogen-containing polydimethylsiloxane by using the formula [17] below:

(1) $$E\%=\frac{B\times W_{ss\;}\times 8}{A\times W_{ms}\times 88}\times 100$$

where *E%* is the content of the H, *W_ss_* is the mass of the standard sample, *W_ms_* is the mass of the measurement sample, *B* is the ^1^H-NMR integral area of the Si–H proton peak of the hydrogen-containing polydimethylsiloxane, and *A* is the ^1^H-NMR integral area of the dioxane.

Fourier transform infrared (FT-IR) spectroscopy was obtained using a Bruker VERTEX70 FT-IR (Bruker, Ettlingen, Germany), with a 2 cm^−1^ resolution with 16 scanning cycles. Surface properties were determined using X-ray Photoelectron Spectroscopy (Axis Ultra DLD, Shimadza-Kratos, Manchester, UK) to measure the different resin surface elements, and the resin’s surface contact angle was measured using a DSA20 video optical contact angle measuring instrument (Dataphysics, Filderstadt near Stuttgart, Germany). Then, Equations (2) and (3) were used to characterize the coating surface energy [15]:

(2) $$\gamma_{sl\;}=\frac{\gamma_{\mathrm{lg}}}{2}(\sqrt{1+\sin^{2}\theta }+\cos\theta ),0\leq \theta \leq \pi$$

(3) $$\gamma_{sg\;}=\frac{\gamma_{\mathrm{lg}}}{2}(\sqrt{1+\sin^{2}\theta }+\cos\theta ),0\leq \theta \leq \pi$$

where *γ_sl_* is the surface energy of the solid and the liquid, *γ_sg_* is the surface energy of the solid and the gas, *γ*_lg_ is the surface energy of the liquid and the gas, and *θ* is the value of the contact angle.

### 2.5. UV Curing Operation

All samples were cured for 40 s and the samples were cured using a UV light source (RW-UVA-200U, Runwing, Taijin, China) at 250–400 nm with an irradiation dose of 40 mW/cm^2^ on the surface of the samples.

## 3. Results and Discussion

### 3.1. Synthesis and Characterization of Novel UV-Curable Fluorinated Siloxane Polymers

These novel UV-curable fluorinated siloxane polymers were synthesized by stepwise reactions of AA, MEHQ, AIBN, AGE, mercaptoethanol, and hexafluorobutyl acrylate (HFOA), dodecafluoroheptyl acrylate (DFOA), and trifluorooctyl methacrylate (TFOMA), following the synthesis route shown in Figure 1.

#### 3.1.1. Characterization of Silicone Intermediates (H-PDMS, OH-PDMS, and EPDMS)

**Figure 2:**
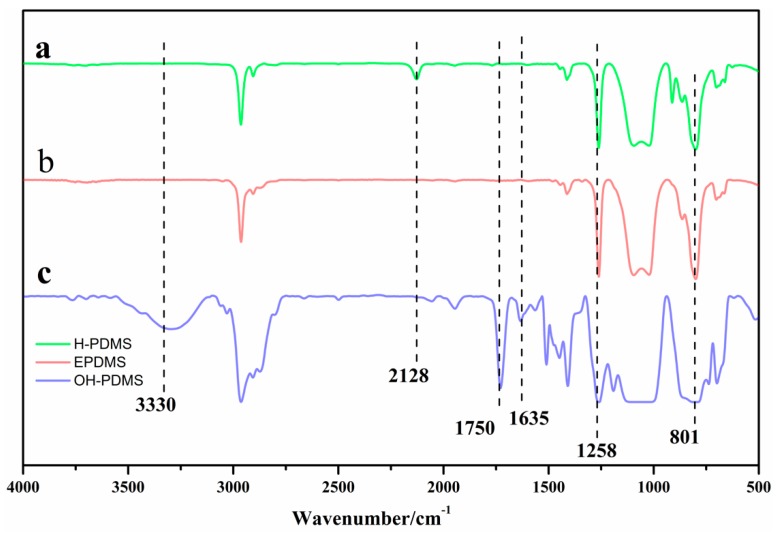
Fourier transform infrared (FT-IR) spectroscopy results of the intermediaries of fluorinated graft copolymer, where a is H-PDMS, b is EPDMS, and c is OH-PDMS.

**Figure 3:**
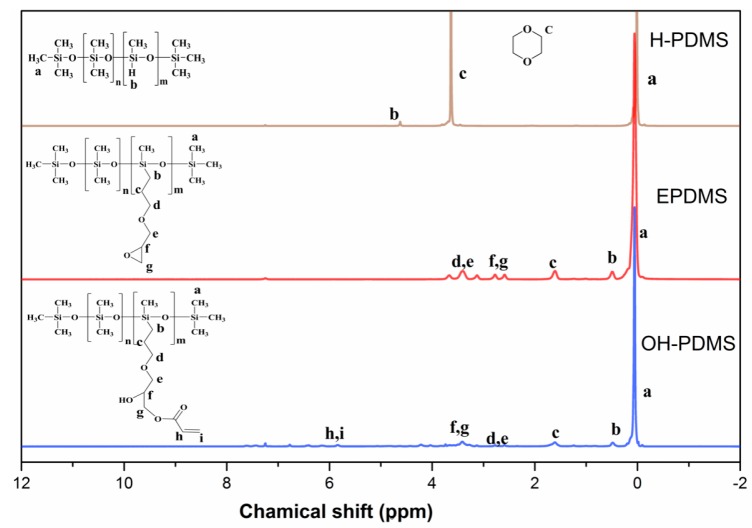
Proton nuclear magnetic resonance (^1^H-NMR) spectra of H-PDMS, OH-PDMS, and EPDMS.

The silicone intermediates of H-PDMS, OH-PDMS, and EPDMS were characterized by FT-IR and ^1^H-NMR, as shown in Figure 2 and Figure 3. We combined Equation (1) with the ^1^H-NMR of H-PDMS to calculate the H content of the hydrogen-containing polydimethysiloxane as 0.25%.

The chemical structures of H-PDMS, EPDMS, and OH-PDMS were confirmed by FT-IR (Figure 2) and ^1^H-NMR (Figure 3). As shown in Figure 2, the wide and dispersive absorption peak around 3330 cm^−1^ demonstrated the presence of the –OH group in EPDMS. In addition, as shown in Figure 3, the appearance of signals at the place of f and g and the peaks around *δ* 2.51–2.80 ppm, which are the methylene peaks belonging to the epoxy. A specific peak appeared around *δ* 2 ppm in the spectrum of OH-PDMS and EPDMS, which can be ascribed to the –OH peak. These peaks showed that the epoxy group at the side-chain of the silicon oil intermediates had opened. The ^1^H-NMR spectrum of OH-PDMS showed the appearance of signals at the place of h and I, and the peaks around *δ* 6.18–5.80 ppm are the C=C peak that belongs to acrylic acid. The results further support our conclusion that EPDMS and OH-PDMS were successfully synthesized.

#### 3.1.2. Characterization of Modified Fluorine-Containing Acrylic Acid

**Figure 4:**
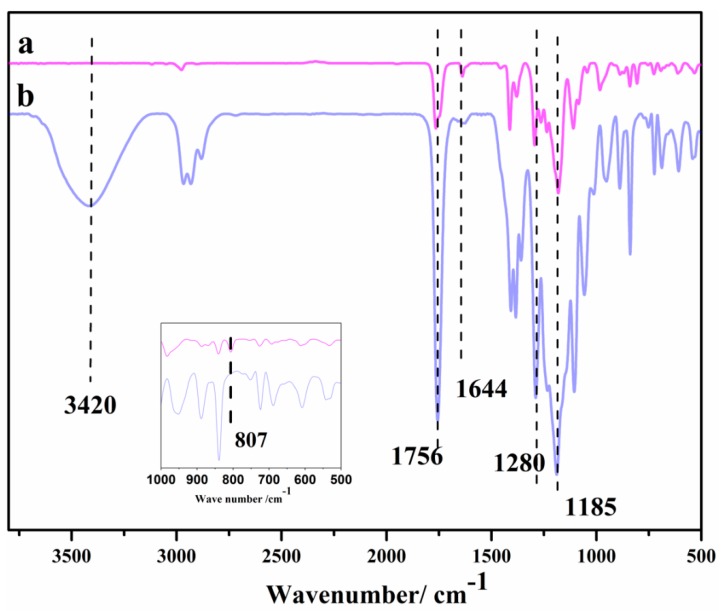
FT-IR of modified fluorine-containing acrylic acid: a, the HFOA; b, the copolymer reacted with mercaptan (OH-G01).

**Figure 5:**
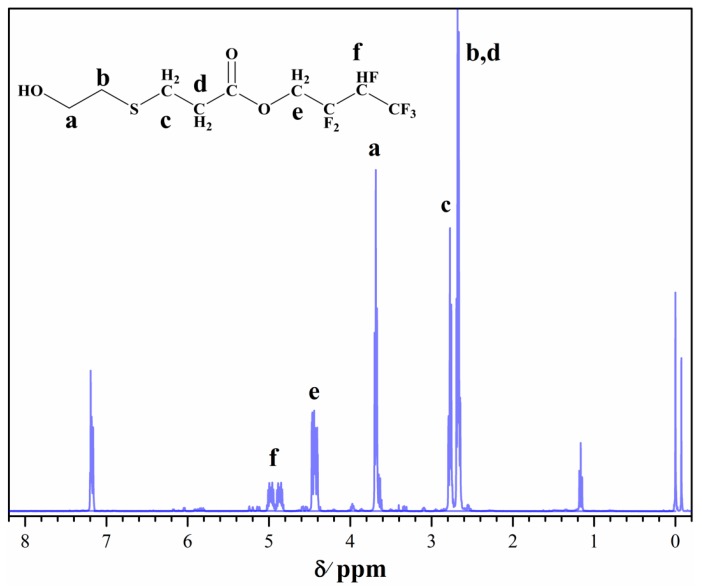
^1^H-NMR spectra of OH-G01.

As shown in Figure 4, the peaks at 1185, 1756, and 1280 cm^−1^ belong to the stretching vibration band of the C–O–C, C=O, and C–F groups, respectively. The wide and dispersive absorption peak at 3024 cm^−1^ belongs to the –OH group, and the peak of the C=C group belongs to the acrylic acid appearing at 1644 and 807 cm^−1^. To further confirm the product, as shown in Figure 5, signals at *δ* 1.16 ppm and *δ* 2.6–2.8 ppm belong to the –OH group and CH_2_–S–CH_2_–CH_2_, respectively. The results indicated that the product was successfully modified by acrylation.

### 3.2. UV-Curing Behavior

**Figure 6:**
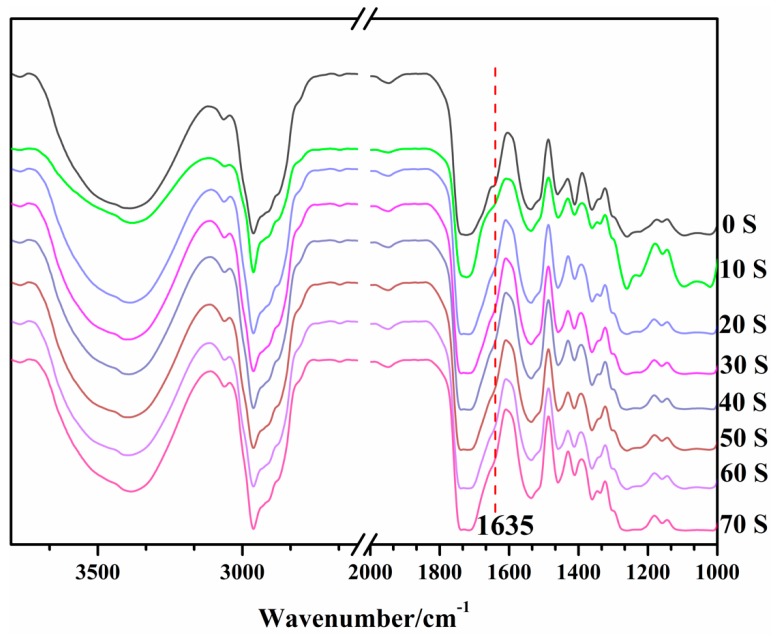
The UV-curable behavior of SG01 as monitored by FT-IR.

The composition of SG01 resin is shown in Table 1. The UV-curing behavior was investigated by analyzing series of FT-IR spectroscopies. As shown in Figure 6, the descent of the peak at 1630 cm^−1^ of C=C, belonging to the acrylic acid stretching vibration bond, was observed after 10 s UV light irradiation. The peak at 1630 cm^−1^ is the indicator for the resin curing process. As the exposure time increased, the peak at 1630 cm^−1^ decreased slowly, until after 40 s, where no further changes occurred. These FT-IR monitoring results clearly show that the coating contained a cross-linked network formed through acrylate polymerization, which prevented the movement of monomers. The results demonstrated that the fluoro-siloxane resin has excellent UV-cured performance.

### 3.3. Surface Characterization

#### 3.3.1. X-Ray Photoelectron Analysis

**Figure 7:**
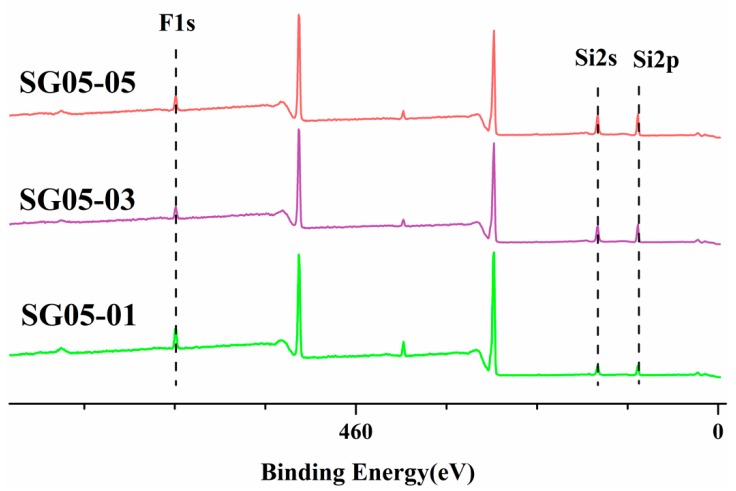
X-ray photoelectron spectroscopy (XPS) of the SG05 series resin at different low-surface energy masses: 0.1 g, 0.3 g, and 0.5 g, denoted by SG05-01, SG05-03, and SG05-05, respectively.

The surface information of fluorinated siloxane UV-cured resin on the air-polymer side was measured by XPS analysis. The peaks at 686, 149, and 99 eV were assigned to F1s, Si2s, and Si2p respectively, as shown in Figure 7 and Table 2. Theoretically, the F element resin content should be 0.148% in SG05 resin. Actually, according to the measurement results, the F element content was much higher than the theoretical value by about 19-fold. Given this result, the data further demonstrate that F has the ability to easily migrate to the surface. Due to the –CF_3_ and –CF_2_ functional groups have low surface energy (surface energy of ~15 mN⋅m^−1^ and ~25 mN⋅m^−1^ respectively), the fluorine groups tended to migrate to the surface of the coatings, and the flexible siloxane also had a good tendency to move, thus the measurement values were higher than the theoretical values. Furthermore, with the content of fluoro-silioxane resin increasing, the surface F content was also augmented up to the saturation value, when the addition reached 0.3 g the F content did not notably change owing to the F enrichment achieving surface saturation and the F content no longer being able to migrate to the surface.

#### 3.3.2. Contact Angle and Surface Energy of the Resin

**Figure 8:**
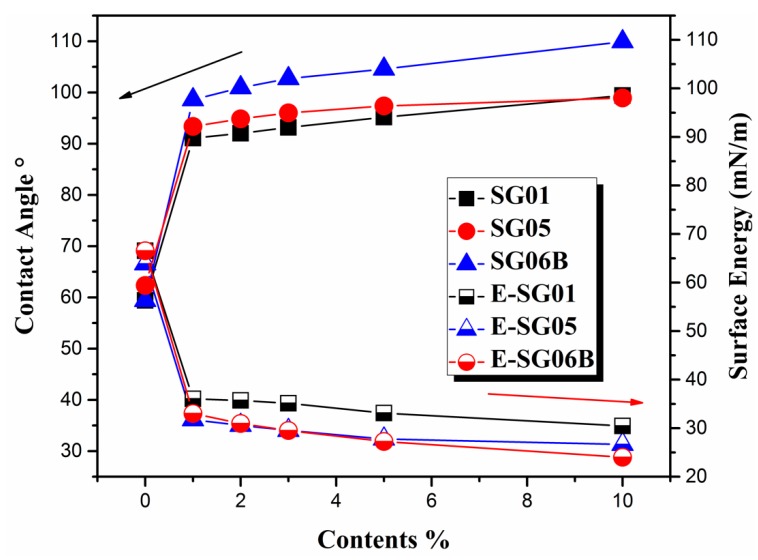
The contact angle and the surface energy of the resins.

**Figure 9:**
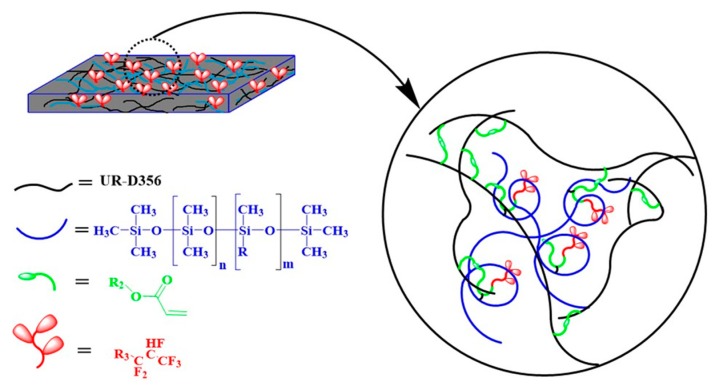
Schematic diagram of the micro-structure stage.

The contact angles were measured to assess the wettability of Si-G01, Si-G05, and Si-G06B modified with DR-U356 resin. As shown in Figure 8, the pure DR-U356 resin contact angle was 59.41°. With the addition of fluorinated siloxane polymers at only 1 wt %, the contact angle increased dramatically up to more than 90°. The reason for this phenomenon is that perfluoroalkyl terminal groups have a good ability to migrate to the surface, whereas flexible siloxane has a tendency to move. Therefore, when the fluorosilicone content is low, there is a significant effect on the contact angle of the resin. Since the fluorine-containing groups and the siloxane have very low surface energy, their enrichment on the surface induced extremely low surface energy and the resin was transformed from the hydrophilic to the hydrophobic state, as illustrated in Figure 9.

As shown in Figure 8, the SG06B series had a higher contact angle compared to the other two series resins. Firstly, the SG06B polymer had more fluorine-containing chains, leading to a more powerful ability to migrate to the surface. Secondly, the SG06B fluorine end group system tended to be out of the system and enter the air interface in an upright shape. Longer fluorine-containing segments can have more fluorine end groups protruding, which indirectly increases the fluorine content per unit area, so SG06B had the highest contact angle. The series of SG01 and SG05 had fewer fluoride chains and some of them are easily enwrapped by the siloxane segment. For the structure of G01 and G05 monomers, G05 has more branched-type fluorine end groups and G01 is a linear fluorine end group; they are different from G06B’s linear fluorine end groups. Most of the G05 fluorine-containing segments were wrapped up in the system. The alkane cannot reach the surface, so the contact angles of the two groups were not much different. As shown in Figure 9, after being subjected to ultraviolet light for about 40 s, the DR-U356 resin system formed a relatively dense crosslinked network structure. Some of the fluorine migrated to the surface of the matrix resin, and the other part was enwrapped inside the matrix resin.

#### 3.3.3. Friction Recovering Properties

**Figure 10:**
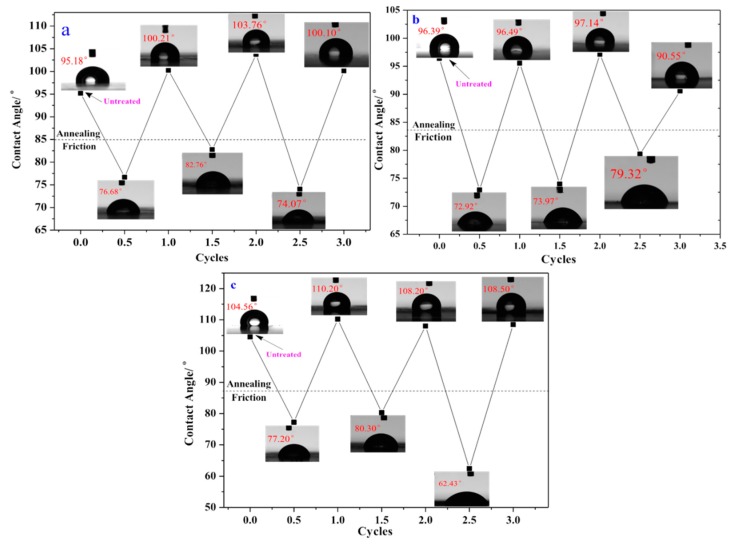
The friction recovering cycle test of the SG01-05 resin (**a**); The friction recovering cycle test of SG05-05 resin (**b**); The friction recovering cycle test of SG06B-05 resin (**c**).

Figure 10a–c demonstrate the friction recovering properties of the SG01-05, SG05-05, and SG06B-05 resins, respectively, examined using friction-annealing progressing. After being rubbed with 600 mesh sand paper about 10 times, the contact angle of the resins decreased below the original values. However, the SG01-05 and SG06B-05 resins, annealed under 120 °C for 30 min, demonstrated higher contact angles than the original values. SG05-05 only attained the original value. Compared to other films [18], SG01-05 and SG06B-05 resins showed excellent performance in terms of self-healing.

The self-healing processing of the low surface energy resins (SG01-05 and SG06B-05) was described as follows: once the SG01-05 and SG06B-05 were rubbed with 600 mesh sandpaper, the F elements on the surface were either destroyed or removed. After SG01-05 and SG06B-05 annealed at 120 °C for 30 min, the inner F elements migrated to the film surface to reduce the surface energy, driven by thermal energy. Even after three cycles of the friction-annealing process, SG01-05 and SG06B-05 maintained their excellent self-healing performance.

### 3.4. Differential Scanning Calorimetry (DSC)

**Figure 11:**
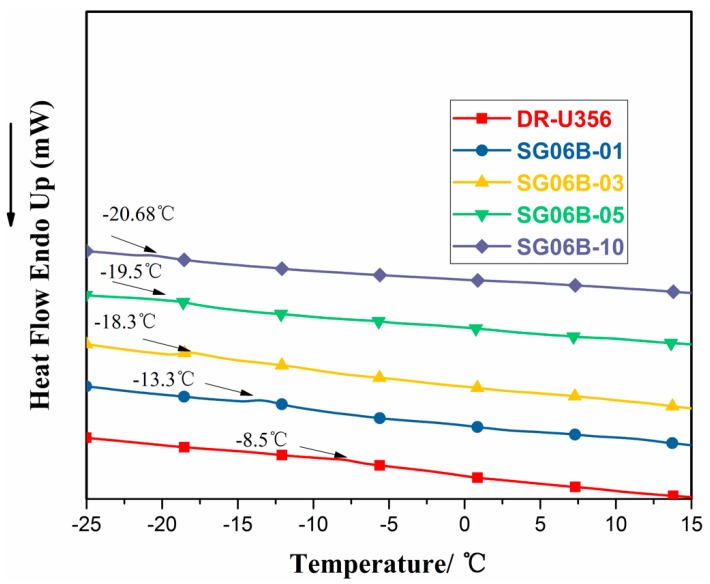
Differential scanning calorimetry (DSC) curves of different contents of SG06B graft UV-cured resin.

The glass transition temperatures (*T_g_*) of the UV-cured resin were investigated by DSC. As shown in Figure 11, compared to the pure DR-U356 resin with the increase in the content of SG06B, the *T_g_* of the resins decreased. This suggests that the incorporation of fluorination destroyed the cross-linked density of pure DR-U356 resin. When the content of SG06B was more than 0.3 wt %, the *T_g_* value decreased. The reason for this was that SG06B-03 had more and longer siloxane spacers than SG06B-01, so some fluorinated groups were buried by siloxane segments, which decreased the negative effect of destroying the crosslinking density of the pure DR-U356 resin. With the increasing SG06B polymer content, the resin system experienced phase segregation due to the poor compatibility between the SG06B polymer and pure DR-U356 resin.

### 3.5. Chemical Resistance

**Figure 12:**
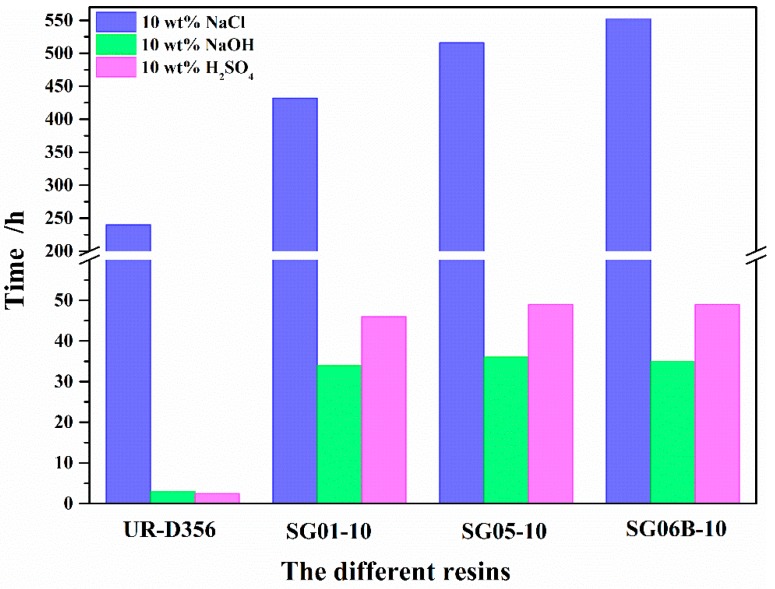
The bubble time of different resins after being dipped in salt, alkaline, and acid solution.

As shown in Figure 12, three series resins added in the amount of one gram had excellent chemical resistance. The resistance to 10 wt % NaOH solution corrosion was up to more than 10 times that of pure UR-D356 resin. The resistance to 10 wt % H_2_SO_4_ solution was 49 h, which is almost 20 times that of pure UR-D356 resin. This phenomenon can be explained as the siloxane and the fluoride chain segment have good inertness, which improves the stabilization of the resins. These results indicate that the addition of the fluoropolymer effectively improves the chemical resistance of the matrix resin.

**Figure 13:**
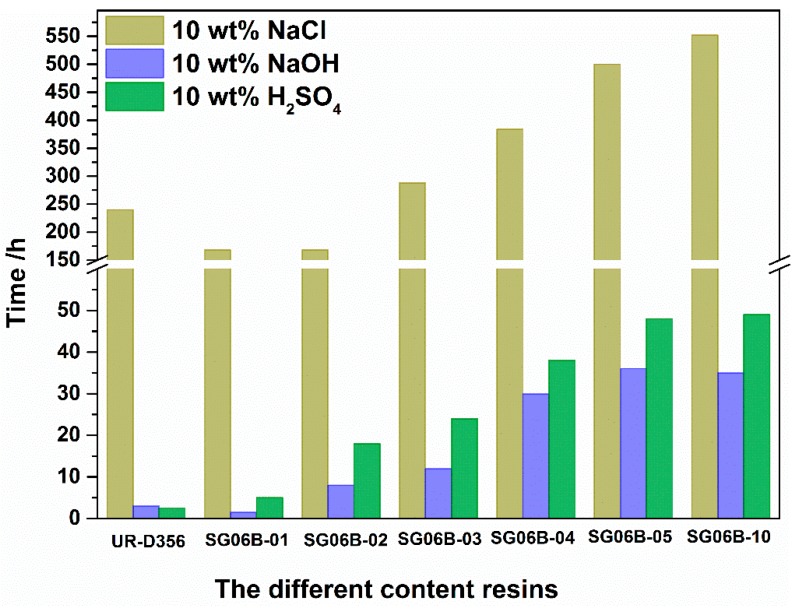
The bubble time with different contents of SG06B series polymers after being dipped in salt, alkaline, and acid solution.

As shown in Figure 13, when the addition was one or two grams, the resin’s resistant destruction ability was weaker than the matrix resin. As part of this phenomenon, as the fluoro-siloxane resin has very low polarity and the polyurethane has very high polarity, they have poor miscibility. Therefore, the resin does not have a pyknotic surface. When the addition was increased to five grams, the chemical resistance notably improved with over 500 h of bubble time.

## 4. Conclusions

In summary, three series of UV-curable fluorinated siloxane polymers (Si-G01, Si-G05, Si-G06B) were successfully synthesized. They were used as surface modifiers of UV-curable commercial polyurethane (DR-U356) at different concentrations (1, 2, 3, 4, 5, and 10 wt % of DR-U356). Hydrophobic states (91°, 92°, and 98°) were obtained at low concentrations (1 wt %). The XPS results indicated that the fluorinated and siloxane groups were liable to migrate to the surface of resins, but with increasing fluoropolymer content, fluorine reached that saturation state on the surface. The incorporation of graft fluorinated siloxane polymers (Si-G01, Si-G05, and Si-G06B) in different amounts into commercial polyurethane (DR-U356) had a significant effect on the chemical resistance behavior of the cured polyurethane, since the fluorinated polymers had good chemical resistance. This work has many wide application prospects, such as water-resistant and anti-fingerprint applications, and provides a new strategy for building low surface energy materials or for corrosion resistant paint.

## Figures and Tables

**Table 1:** The composition of graft fluorinated siloxane resins.

	Si-G01 (g)	Si-G05 (g)	Si-G06B (g)	DR-U356 (g)	Acetone (g)	Initiator (g)
SG01-01	0.1	×	×	9.9	2.0	0.4
SG01-02	0.2	×	×	9.8	2.0	0.4
SG01-03	0.3	×	×	9.7	2.0	0.4
SG01-04	0.4	×	×	9.6	2.0	0.4
SG01-05	0.5	×	×	9.5	2.0	0.4
SG01-10	1.0	×	×	9.0	2.0	0.4
SG05-01	×	0.1	×	9.9	2.0	0.4
SG05-02	×	0.2	×	9.8	2.0	0.4
SG05-03	×	0.3	×	9.7	2.0	0.4
SG05-04	×	0.4	×	9.6	2.0	0.4
SG05-05	×	0.5	×	9.5	2.0	0.4
SG05-10	×	1.0	×	9.0	2.0	0.4
SG06B-01	×	×	0.1	9.9	2.0	0.4
SG06B-02	×	×	0.2	9.8	2.0	0.4
SG06B-03	×	×	0.3	9.7	2.0	0.4
SG06B-04	×	×	0.4	9.6	2.0	0.4
SG06B-05	×	×	0.5	9.5	2.0	0.4
SG06B-10	×	×	1.0	9.0	2.0	0.4

**Table 2:** Comparison of theoretical and measured amounts of SG05 series.

	Theory (%)	Measurement (%)
**SG05-01**	0.148	2.86
**SG05-03**	0.444	2.21
**SG05-05**	0.740	2.79
